# Warping an atlas derived from serial histology to 5 high-resolution MRIs

**DOI:** 10.1038/sdata.2018.107

**Published:** 2018-06-19

**Authors:** Stephanie Tullo, Gabriel A. Devenyi, Raihaan Patel, Min Tae M. Park, D. Louis Collins, M. Mallar Chakravarty

**Affiliations:** 1 Integrated Program in Neuroscience, McGill University, Montreal, Canada; 2 Computational Brain Anatomy Laboratory, Cerebral Imaging Centre, Douglas Mental Health University Institute, Montreal, Canada; 3 Department of Psychiatry, McGill University, Montreal, Canada; 4 Department of Biological and Biomedical Engineering, McGill University, Montreal, Canada; 5 Schulich School of Medicine and Dentistry, Western University, London, ON, Canada; 6 McConnell Brain Imaging Centre, Montreal Neurological Institute, Montreal, Canada

**Keywords:** Magnetic resonance imaging, Brain imaging, Brain, Computational neuroscience

## Abstract

Previous work from our group demonstrated the use of multiple input atlases to a modified multi-atlas framework (MAGeT-Brain) to improve subject-based segmentation accuracy. Currently, segmentation of the striatum, globus pallidus and thalamus are generated from a single high-resolution and -contrast MRI atlas derived from annotated serial histological sections. Here, we warp this atlas to five high-resolution MRI templates to create five de novo atlases. The overall goal of this work is to use these newly warped atlases as input to MAGeT-Brain in an effort to consolidate and improve the workflow presented in previous manuscripts from our group, allowing for simultaneous multi-structure segmentation. The work presented details the methodology used for the creation of the atlases using a technique previously proposed, where atlas labels are modified to mimic the intensity and contrast profile of MRI to facilitate atlas-to-template nonlinear transformation estimation. Dice’s Kappa metric was used to demonstrate high quality registration and segmentation accuracy of the atlases. The final atlases are available at https://github.com/CobraLab/atlases/tree/master/5-atlas-subcortical.

## Background and Summary

The subcortical structures of the brain, such as the striatum and thalamus, are positioned deep below the cortical manifold and serve as important network hubs and relays. Amongst their many roles in brain function, these regions are critically involved in motor function, addiction, and mood regulation^1–3^. Further, these structures have multiple reciprocal connections between one another and to regions implicated in complex cognitive tasks such as the frontal cortex^3,4^, and are amongst the last to mature (as indexed by age of peak volume attainment) through the course of normal development^5,6^. Despite their importance in daily functioning and implication in neuropsychiatric disorders (such as schizophrenia^7–9^ and geriatric depression^10,11^) and in movement disorders (such as Parkinson’s disease and Huntington's diseases), these regions are under-studied, in part due to the paucity of methods available for accurate visualization and analysis of in vivo magnetic resonance imaging (MRI)^7,12^.

The gold standard for neuroanatomical MR image segmentation is manual delineation by an expert human rater based on prior histological and functional data. However, with the availability of increasingly large MRI datasets^13–15^, the time and expertise required for manual segmentation becomes prohibitive. Automated segmentation techniques, methodologies where expertly manually labelled MRI atlases are warped to target MR images using nonlinear registration methods, are thus actively used in neuroimaging experiments, given that these methods are reliable, objective, and reproducible^7,16^.

Given the proliferation of automated segmentation tools, a recent study by our group^7^ sought to establish the validity and reliability of segmentation methods in defining the striatum, globus pallidus, and thalamus using FreeSurfer^17^ (http://surfer.nmr.mgh.harvard.edu), FSL-FIRST^18^ (http://fsl.fmrib.ox.ac.uk), and MAGeT-Brain^16,19^ (https://github.com/CobraLab/MAGeTbrain). When comparing manually defined labels to labels generated using each segmentation tool on thirty subjects (15 patients with first episode psychosis and 15 controls), correlations between automated and manually segmented volumes were strongest for MAGeT-Brain^7^.

The current implementation of the MAGeT-Brain pipeline allows for subject-based segmentation of the hippocampal subfields^16,20^, hippocampal white matter^21^, cerebellar lobules^22^, and the striatum, globus pallidus, and thalamus^23^. For hippocampal and cerebellar segmentation, five atlases, created from manual segmentation on five high-resolution MRI templates, are used to derive subject-based segmentation^20–22^. However, segmentation of the striatum, globus pallidus, and thalamus are derived from a single input atlas, which was derived from reconstructed histological data warped onto the Colin27 MRI template^23^. As the basal ganglia and thalamus atlas was created long before our more recent atlases, the resulting difference in input atlas source requires two separate executions of MAGeT-Brain. Thus, reproducing segmentation of the striatum, globus pallidus and thalamus in the same five MRI templates would homogenize our input atlases and accordingly streamline future work-flows, allowing simultaneous multi-structure segmentation.

Moreover, many groups^24–30^, including our own^7,16,19,22,31^, have shown that multiple atlases improve overall segmentation reliability over model-based single atlas approaches. This is specifically true when using MAGeT-Brain which was designed to facilitate segmentation using a small number of hard-to-define input atlases. Although our group demonstrated reliable segmentation of the striatum, pallidum, and thalamus using a single atlas^19,32^, we have also shown that multi-atlas segmentation improves segmentation accuracy (and has been seen to plateau) with the inclusion of up to five input atlases, for hippocampal subfield segmentation^16^, and improved segmentation accuracy for the basal ganglia and thalamus with the inclusion of three MRI templates as input atlases^19^.

In this paper, we detail the steps used to adapt an existing high-resolution atlas of the basal ganglia and thalamus^23^ to match five high-resolution MRIs using atlas-to-template warping techniques previously described by our group^23,32^. The creation and use of five de novo atlases of the striatum, globus pallidus and thalamus, along with three-dimensional surface rendering of these structures will serve as a new set of priors for input into an existing multi-atlas segmentation pipeline, MAGeT-Brain^19^, with the primary benefit of homogenizing and improving upon its current implementation. MAGeT-Brain with the five newly-created input atlases will provide improved subject-based automated identification and estimation of volume and shape of striatum, globus pallidus and thalamus, and allow for the investigation of subcortical morphology in various subject groups ranging from healthy individuals to patient populations.

## Methods

### Atlas-to-template warping technique

We present a continuation of work previously presented in Chakravarty *et al.* (2006), which describes the creation of an atlas of the basal ganglia and thalamus derived from serial histological data, detailing 108 structures^23^. The brain used to create the histological data set was acquired in 1957 from a male patient who died of non-neurological causes at the Montreal Neurological Institute/Hospital, in which is intensively studied and used for teaching over the past 45 years. The histological images were manually segmented and labeled combining information and nomenclature using three different references^33–35^. Each image has a center-to-center voxel spacing of 0.034 mm×0.034 mm and a slice-to-slice spacing of 0.7 mm. Serial histological sections were reconstructed into a contiguous 3D volume using a slice-to-slice nonlinear registration technique which minimizes anatomic mis-registration throughout the reconstructed data set and an intensity correction scheme which analyzes local neighborhoods on each slice in order to build a voxel-by-voxel multiplicative field to correct for local variations in image intensities between slices. The reconstructed volume was registered to a standard MRI data set using a novel atlas-to-template warping technique, described in Chakravarty *et al.* (2006, 2008). This methodology is described in detail in previous work^23,32^ and was validated against manual segmentation^23,36^, intraoperative recordings^32^, and functional MRI activations^37,38^.

High-resolution T1-weighted images used for the creation of five subcortical atlases were acquired from 5 healthy subjects (2 male, 3 female, aged 29–57, average age of 37 years)^20^. All images were acquired on a 3 T GE Discovery MR 750 system (General Electric, Milwaukee, WI) at the Centre for Addiction and Mental Health (CAMH) in Toronto, Canada using an 8-channel head coil. Three sets of high-resolution T1-weighted images were acquired from each subject. T1-weighted images were acquired using the 3D inversion-prepared fast spoiled gradient-recalled echo acquisition (FSPGR-BRAVO; TE/TR=4.3 ms/9.2 ms, TI=650 ms, α=8°, 2NEX, FOV=22 cm, slice thickness=0.6 mm, 384×384 in-plane steps) with an isotropic voxel size of 0.6 mm. A final isotropic voxel size of 0.3 mm was obtained using zero-filling reconstruction filters, ZIPX2 and ZIP512, done on the GE scanner. For each subject, the three T1-weighted images were each corrected for RF inhomogeneity non-uniformity^39^ and normalized to a fixed intensity range (0–10,000) on a voxel-by-voxel basis to enhance signal and contrast^40^ to ultimately produce one final T1-weighted image volume. The final T1-weighted image volume was produced from the average of the three corrected MR images for each subject using a rigid-body alignment^41^. All images were converted to the MINC file format and subsequent image processing and neuroanatomical labeling was performed using tools from the MINC software distribution (http://www.bic.mni.mcgill.ca/ServicesSoftware/HomePage). The data acquisition was approved by the Centre for Addiciton and Mental Health Research Ethics Board, and all subjects provided written, informed consent for data acquisition and sharing. These templates were previously used by our group for the creation of atlases of the hippocampal subfields^16,20^, hippocampal white matter^21^, and the cerebellum^22^.

**Figure 1:**
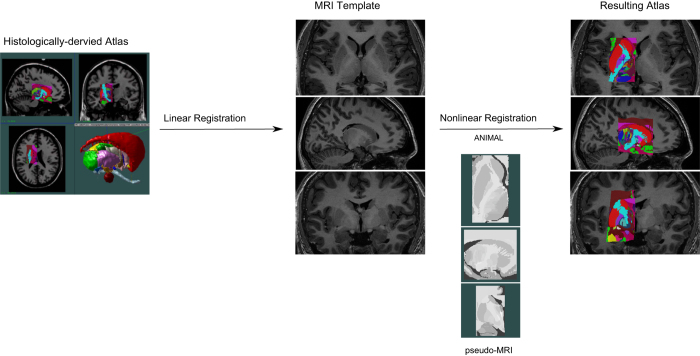
Outline of the workflow of the atlas-to-template customization. Atlas of the striatum, globus pallidus, and thalamus derived from a histologically-derived atlas (Chakravarty *et al.*, 2006) was warped to each of the five high-resolution T1-weighted MRI reference brain scans using linear registration, pseudo MRI creation, and nonlinear registration.

The atlas-to-template warping technique used here for the creation of five atlases was initiated with linear registration of the histologically-derived atlas to five T1-weighted high-resolution MRI templates^20^ (as described in Chakravarty *et al.* (2006)). By identifying homologous landmark-pairs, linear transformations are estimated to map the atlas to each MRI using a 12-parameter transformation (3 of each of translations, rotations, scales, and shears). After accounting for global differences with the linear registration, the remaining morphological differences between the atlas and the templates were accounted for using nonlinear registration to further refine the fit between the atlas and each template. However, the inherent differences in contrast and morphology presents a challenge for customization of the histology-derived atlas to MRI templates. To account for these differences, pseudo-MRIs were created by manually assigning an intensity value to each label value of the atlas based on the intensity of the matching structure in MRI template for which it is registered to. This remapping of the atlas labels to match the intensity profile of the templates allows the nonlinear registration algorithm to treat the atlas as a standard input MRI. Figure 1 details an overview of the atlas-to-template warping technique, from linear transform to pseudo-MRI to final fit.

The nonlinear registration was performed using the automatic nonlinear image matching and automatic labeling algorithm (ANIMAL)^42,43^ to register the pseudo-MRIs to the MRI templates. This nonlinear transformation is estimated in a hierarchical fashion, where large deformations are estimated using volumes blurred with a Gaussian kernel with a large full-width at half maximum (FWHM), which gets progressively smaller to refine deformations, to optimize a transformation that maximizes the similarity between the source and target image. The final nonlinear transformation is represented by a deformation field (defined by vectors spaced on a grid representing a three-dimensional translation at each node of the lattice grid) that is iteratively estimated in a two step process; the first step involves the calculation of local translations for each node that optimizes a local objective function (the correlation coefficient) and the second is a regularization step to ensure that the deformation field is continuous. The definition of the final deformation field produced by ANIMAL’s two-step algorithm is dependent upon the step size (the spacing between the lattice points of the grid), sublattice diameter (the diameter of a local spherical neighbourhood around each node in which the deformation is estimated) and the sublattice (number of nodes contained within a local spherical neighbourhood)^32,40,42^. Additionally, three regularization parameters are used to improve the quality of the nonlinear transformation estimated, where the weight parameter determines the proportion of each local translation estimated at each iteration that will be used at the next iteration, the stiffness parameter is the smoothing factor between iterations (to ensure continuity), and the similarity-cost ratio parameter balances the similarity between the volumes with the cost of the transformation^32,40,42,43^. The parameters used to calculate the atlas-to-template transformation using ANIMAL are given in Table 1. The final transformation was achieved through an optimization of a correlation coefficient objective function, and described in Collins & Evans (1997) and Chakravarty *et al.* (2006, 2008). Other nonlinear registration algorithms were attempted, and are discussed in section “Nonlinear Registration Algorithms”.

### Atlas warping evaluation

The atlas-to-template warping technique presented in section “Atlas-to-template warping technique” was evaluated against manual segmentation of the striatum, globus pallidus, and thalamus to validate the accuracy of the atlas labels before using these atlases as input to the MAGeT-Brain pipeline.

### Dice’s Kappa Overlap Metric

The labels defined in the atlas were compared to manual segmentation to determine the accuracy of the atlas-to-template warping technique. Dice similarity coefficient (also referred to as Dice's Kappa) (κ) metric is used here to evaluate the quality of the overlap of the atlas labels derived from the atlas-to-template warping technique with manual segmentation, as this segmentation method is the current gold-standard method. The Dice’s Kappa overlap metric score is determined by:

κ=2a/(2a+b+c)

where a is the number of voxels common to both methods of segmentation, and b+c is the sum of the voxels uniquely labeled by each segmentation method. A higher Kappa value denotes a higher degree of overlap, where a score of 0 represents no overlap and a value of 1 represents perfect overlap, and scores greater than 0.7 are deemed acceptable in the segmentation literature^32,44–46^.

### Manual Segmentations

To evaluate the ‘‘goodness of fit” of atlas labels, segmentations of the striatum, globus pallidus, and thalamus, were generated using MAGeT-Brain segmentation^19^ using the current single input atlas by one of the authors (M.T.M.P.) to obtain labels on the five high-resolution T1 weighted images. Subsequently, these labels were manually corrected, particularly in regions where the endogenous contrast boundaries are not as distinct. These semi-manual segmentation were then used to evaluate segmentation accuracy of the atlas labels.

### Surface Representations

**Figure 2:**
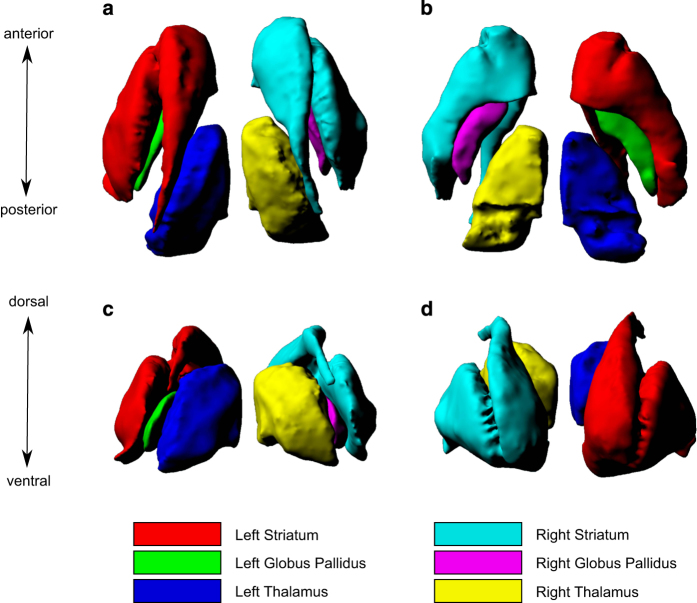
Three dimensional reconstruction of high-resolution striatum, globus pallidus and thalamus atlases. The five subcortical atlases were averaged to obtain a model atlas in which was used to derive surface representations of the striatum, globus pallidus and thalamus. (**a**) presents a superior view, (**b**) presents an inferior view, (**c**) presents a posterior view and (**d**) presents an anterior view of the bilateral striatum, globus pallidus and thalamus.

A single surface-based representation of the striatum, globus pallidus and thalamus are defined on a model atlas. The model was created using atlas-creation methods described previously^47–49^. Briefly, one of the five MR templates is selected as the target, and the other four templates are registered in a 6-parameter (3 translations and 3 rotations) linear registration to this target (carried out using ANTs for MINC formatted images). These images are then registered to each other on a pairwise basis using a 12-parameter linear registration (3 translations, 3 rotations, 3 scales, and 3 shears), and resampled to normalize each image for average linear brain size. The resampled images are then averaged to create an initial model (M0). Next, the resampled images are non-linearly registered to M0 using ANTs, resampled again, and averaged to create M1. This step is repeated twice more, with each additional step improving the accuracy of the model in representing the mean anatomy of the five original atlases^50^. This model atlas provides an averaged neuroanatomical representation of the five atlases for the striatum, globus pallidus and thalamus. The model atlas labels were generated using a majority voting label fusion technique. This atlas has superior contrast, signal, and definition when compared to a single atlas, and most importantly provides a common space for analysis of surface-based metrics generated by the MAGeT-Brain pipeline^50^. Using this model atlas, the striatum, globus pallidus and thalamus were extracted as objects using the marching cubes algorithm in the Display software (part of the MINCtools package, http://www.bic.mni.mcgill.ca/ServicesSoftware/HomePage) to obtain a 3D triangular mesh for each structure bilaterally. These meshes were subsequently manually smoothed using the MeshLab open source system for processing and editing 3D triangular meshes^51^. After manual smoothing, the objects were re-meshed at approximately 0.3 mm vertex-to-vertex spacing on the surface to match the voxel size of the atlases (and consequently, matching the deformation grids from MAGeT-Brain), and an additional quadratic space smoothing was performed^52^. The resulting surfaces have approximately 13000 vertices per striatum, 3000 vertices per globus pallidus, and 6500 vertices per thalamus. The model atlas with surface representations of the striatum, globus pallidus, and thalamus are shown in Figure 2.

The surface representations of the striatum, globus pallidus and thalamus were created to obtain surface-based metrics, including surface area and displacement for shape analysis, using the MAGeT-Brain pipeline. This methodology is described in detail in previous work by our group^6,7,16,31^.

### Code availability

The atlases were created using tools from the MINC software distribution (http://www.bic.mni.mcgill.ca/ServicesSoftware/HomePage); however, NIfTI format (https://nifti.nimh.nih.gov/pub/dist/src/niftilib/nifti1.h) is also availble on our Figshare repository (Data Citation 1). The conversion from MINC to NIfTI-1 format was performed using mnc2nii (part of the MINCtools package: http://www.bic.mni.mcgill.ca/ServicesSoftware/HomePage).

The code for creation of the atlases is accessible through our Figshare repository (Data Citation 1). The ANIMAL script details the commands for the nonlinear registration step of the atlas-to-template warping technique. The resulting atlases contain 108 subcortical structures, all of the structures present in the histologically-derived atlas^23^ (Data Citation 1). The final atlases of the striatum, globus pallidus and thalamus, in which will be used as input atlases for the automated segmentation pipeline MAGeT-Brain, were generated using the mask script, also available in our Figshare repository (Data Citation 1).

MAGeT-Brain (https://github.com/CobraLab/MAGeTbrain), the automated segmentation pipeline, for which these atlases will be used as input to derive subject-based segmentation of the striatum, globus pallidus, and thalamus, is supported on the BIDS app (https://github.com/BIDS-Apps/MAGeTbrain) and OpenNeuro (reproducibility.stanford.edu/openneuro-app-highlights-maget-brain/), in addition to being available as an open source tool from our website (http://cobralab.ca/software/MAGeTbrain/).

## Data Records

The final customized atlases and surface representations of the striatum, globus pallidus, and thalamus, as well as the five MR templates can be retrieved from the Figshare repository (Data Citation 1) and is available in both MINC (http://www.bic.mni.mcgill.ca/ServicesSoftware/MINC) and NIfTI-1 (https://nifti.nimh.nih.gov/pub/dist/src/niftilib/nifti1.h) format.

Moreover, these files are also located on our GitHub repository as these atlases and surface representations are to be used with MAGeT-Brain (http://cobralab.ca/software/MAGeTbrain/). The atlas labels and surface representations for the striatum, globus palidus and thalamus can be found at https://github.com/CobraLab/atlases/tree/master/5-atlas-subcortical, while the five high-resolution MRI templates can be found here (https://github.com/CobraLab/atlases), along with our other atlases created using the same MRI templates, namely our hippocampal subfields and white matter atlases (Winterburn *et al.*, 2011, Pipitone *et al.*, 2014 & Amaral *et al.*, 2016), as well as the cerebellar atlases (Park *et al.*, 2014).

As alluded to above, the atlas resulting from the atlas-to-template warping technique delineates 108 subcortical structures however, the final atlases in which will be used as input to the MAGeT-Brain pipeline consist of only the striatum, globus pallidus and thalamus, as these structures were validated for their accuracy, using the Dice’s kappa measures. Due to limited contrast and delineation of certain structures included in the histologically-derived atlas, such as the thalamic nuclei subdivisions, the accuracy of the segmentation for all 108 structures were not validated, as we do not have a gold-standard to compare the automated labels to (due to the lack of manual segmentation protocols for these structures). Nonetheless, the full atlas is available to download, however the accuracy of the automated segmentation for all 108 structures have not been assessed and thus these labels should be used at the discretion of the reader (Data Citation 1). Moreover, neither atlas versions (the 3 structures versus the 108 structure versions) parses the striatum into the caudate nucleus, putamen and nucleus accumbens. Partial volume effects and lack of contrast between the subdivisions of the striatum (limited contrast to delineate the border between the caudate nucleus and nucleus accumbens, as well as the fine-scale cell bridges between the caudate and the putamen) were the main factors for providing a single label for the entire striatum. Any attempt to subdivide the striatum further would be based on heuristic definition.

## Technical Validation

### Atlas-to-template warping technique validation

**Figure 3:**
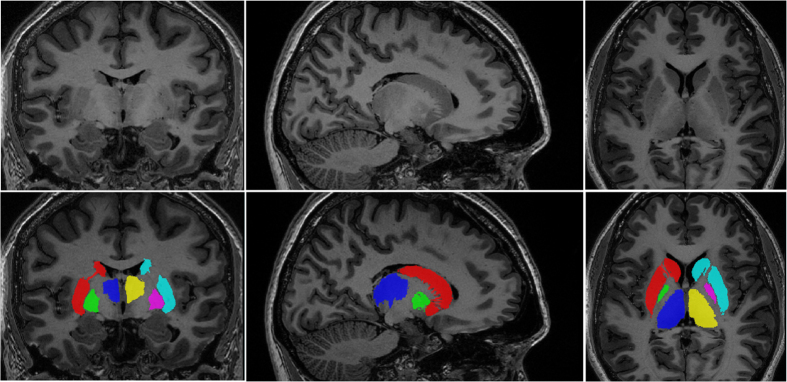
Final Atlas. One of the five subcortical atlases, which will be used as input for MAGeT-Brain, is shown above detailing the three subcortical structures of interest (the striatum, globus pallidus and thalamus) on both hemisphere.

Given that manual segmentation is the current gold standard for anatomical delineation, the manual segmentations mentioned in section “Manual Segmentations” were used to evaluate the “goodness-of-fit” of the atlas labels generated using our atlas-to-template warping technique. All labels of the atlas had high overlap scores (κ>0.83 for all structures bilaterally). Average Dice’s Kappa scores across all five atlases show strong overlap between automated and manual segmentations for the striatum (mean left κ=0.885; right κ=0.882), the globus pallidus (mean left κ=0.836; right κ=0.829) and the thalamus (mean left κ=0.903; right κ=0.899). Average Dice’s Kappa scores across all atlases are shown in Table 2, and Dice’s Kappa scores for each atlas are shown in Table 3. See Figure 3 for an example of the final segmentations of striatum, globus pallidus and thalamus for one of the five atlases.

Based on the Dice’s Kappa scores obtained, the resulting segmentation of the striatum, globus pallidus and thalamus from the atlas-to-template warping is quite accurate. While all Dice’s Kappa scores obtained are accurate by segmentation literature standards, nonetheless, a possible explanation for Dice’s Kappa scores observed for the globus pallidus being lower than those in the striatum and thalamus is the limited resolution and contrast of certain of these structures in MRI data. Compared to the histologically-derived atlas, in which the resolution allows for the posterior portions of the subcortical structures to be well identified, there is limited resolution of these boundaries in MR images^32^. The limited contrast of the pallidal borders may be discrepant with manual segmentation protocols^7,53^, which attests to the challenges in its delineation, and will inevitably result in lower Dice’s Kappa overlap scores. Given the limited contrast for the pallidum in T1w images, the atlas-to-template warping technique was originally performed on high-resolution T2w MR templates however, the Dice’s kappa measures (striatum: left κ=0.803, right κ=0.817; globus pallidus: left κ=0.736, right κ=0.747; thalamus: left κ=0.811, right κ=0.809) were not nearly as high as those obtained when warping the histologically-derived atlas to T1w MR templates (Table 1). Thus, given the improved accuracy of the atlas labels produced on the T1w images, the final atlases described in the paper were derived on T1w MR templates. Another possible improvement of segmentation accuracy in the newly-created atlases could be the use of several sets of serial histological data from multiple atlases. However, multiple large section histology (as the one used as inputs here) are notoriously difficult to achieve. Thus, we chose to use the histological dataset detailed in Chakravarty *et al.*, (2006), given that this data was well studied and delineated by a neurosurgeon from the Montreal Neurological Institute.

### Nonlinear Registration Algorithms

The ANIMAL algorithm^42,43^ was used for the nonlinear registration of the histologically-derived atlas to the five MRI templates, which deforms a source MRI volume, the pseudo-MRI atlas, to match a target MRI volume, the MRI template. Alternate nonlinear registration algorithms were also examined. Based on the results from Klein *et al.*^54^ (2009), where 15 registration algorithms were evaluated based on overlap measures of manually labeled anatomical regions, two other of nonlinear registration algorithms were used for the nonlinear registration step of the atlas-to-template warping. Given that a symmetric normalization (SyN) nonlinear transformation delivered consistently high accuracy across subjects and label sets in Klein *et al.* (2009), two iterations of the SyN transformation, using two different adaptations of Advanced Normalization Tools (ANTS) registration suite^55^, mincANTS and antsRegistration, were implemented. However, neither implementation produced superior registrations to those of ANIMAL, as evidenced by Dice’s Kappa scores (data not shown). In keeping with the methodology for atlas-to-template warping described in Chakravarty *et al.* (2006), ANIMAL was used to nonlinearly register the pseudo-MRIs to the MRI templates to finalize the atlas-to-template warping. The comparison of nonlinear warping techniques for atlas-to-template MRI warping is beyond the scope of this manuscript.

Moreover, two separate ANIMAL runs were performed using different parameters; the first using the parameters described in Chakravarty *et al.* (2006) and a second from Chakravarty *et al.* (2008), where the resolution of the deformation field begins at a higher resolution in the latter paper; that is, the sublattice (the number of nodes) and the sublattice diameter (the diameter of a local spherical neighbourhood around each node) is smaller for the first step of the nonlinear algorithm (sublattice=8; sublattice diameter=10 in Chakravarty *et al.* (2006) whereas sublattice=6; sublattice diameter=8 in Chakravarty *et al.* (2008)). When using the parameters described in Chakravarty *et al.* (2008), the atlases generated had higher Dice’s Kappa scores, compared to the Dice’s Kappa obtained when using the Chakravarty *et al.* (2006) parameters. Given that the resolution of the five MRI templates (0.3 mm^3^ isotropic) onto which the histologically-derived atlas was warped is higher than the resolution of the Colin27 template (1.0 mm^3^ isotropic) used in Chakravarty *et al.* (2006), increasing the resolution of initial step of ANIMAL increased Dice’s Kappa scores. That is, the automated labels generated using the parameters described in Chakravarty *et al.* (2008) for the ANIMAL nonlinear algorithm (as described in Table 1) allows for greater accuracy in capturing the neuroanatomical variation present between the histologically-derived atlas and the MRI templates, and so, these parameters were used for the creation of the five subcortical atlases.

Recent work by Xiao *et al.*^56–58^ established a multi-contrast registration framework that non-rigidly deforms the histologically-derived atlas used here (ref. 23) to MRI data of multiple contrasts (such as T1w, T2*w and T2w images) to obtain segmentations of the subthalamic nucleus, substantia nigra, and red nucleus. While this technique improves accuracy of the segmentations for these nuclei, since using multiple MRI contrasts allows for enhanced visualization of these nuclei compared to the sole T1w contrast, the use of multi-contrast registration is beyond the scope of this manuscript.

## Additional information

**How to cite this article**: Tullo S. *et al*. Warping an atlas derived from serial histology to 5 high-resolution MRIs. *Sci. Data* 5:180107 doi: 10.1038/sdata.2018.107 (2018).

**Publisher’s note**: Springer Nature remains neutral with regard to jurisdictional claims in published maps and institutional affiliations.

## Supplementary Material



## Figures and Tables

**Table 1:** ANIMAL parameters used for atlas-to-template transformation.

Step	Step Size (mm)	Sublattice diameter	Sublattice
1	4	8	6
2	2	6	6
3	1	6	3

**Table 2:** Average Dice’s Kappa scores across all atlases: reliability analysis of the segmentation accuracy of the atlas-to-template warping technique.

	Left hemisphere	Right hemisphere	Average
Striatum	0.885	0.882	0.884
Globus Pallidus	0.836	0.829	0.833
Thalamus	0.903	0.899	0.901

**Table 3:** Dice’s Kappa scores for each MRI template: reliability analysis of the segmentation accuracy of the atlas-to-template warping technique.

	Left hemisphere	Right hemisphere	Average
**BRAIN 1**			
Striatum	0.899	0.886	0.893
Globus Pallidus	0.846	0.838	0.842
Thalamus	0.916	0.899	0.907
**BRAIN 2**			
Striatum	0.887	0.89	0.888
Globus Pallidus	0.833	0.828	0.83
Thalamus	0.904	0.903	0.903
**BRAIN 3**			
Striatum	0.879	0.878	0.878
Globus Pallidus	0.809	0.819	0.814
Thalamus	0.895	0.892	0.893
**BRAIN 4**			
Striatum	0.884	0.882	0.883
Globus Pallidus	0.843	0.827	0.835
Thalamus	0.906	0.904	0.905
**BRAIN 5**			
Striatum	0.879	0.874	0.876
Globus Pallidus	0.851	0.831	0.841
Thalamus	0.895	0.897	0.896
